# 

**Published:** 1875-08

**Authors:** 


FOUR DOLLARS A YEAR, POSTAGE FREE.

VOL. XXXII. August, 1875. NUMBER 8.

S.G.O. INDEXED

### THE Chicago Medical Journal

J. ADAMS ALLEN, M.D., LL.D., WALTER HAY, M.D.,

EDITORS.

ISSUED TWELVE TIMES A YEAR.

CHICAGO:

W. B. KEEN, COOKE & CO., Publishers,

NOS. 113 AND 115 STATE STREET.

